# A new monster from southwest Oregon forests: *Cryptomaster behemoth* sp. n. (Opiliones, Laniatores, Travunioidea)

**DOI:** 10.3897/zookeys.555.6274

**Published:** 2016-01-20

**Authors:** James Starrett, Shahan Derkarabetian, Casey H. Richart, Allan Cabrero, Marshal Hedin

**Affiliations:** 1 Department of Biology, 5500 Campanile Drive San Diego State University, San Diego, CA 92182, USA; 2 Department of Biology, 900 University Avenue, University of California, Riverside, Riverside, CA 92521, USA

**Keywords:** Short-Range Endemic, DNA barcoding, cryptic species, Bayes Factor Delimitation, genealogical congruence

## Abstract

The monotypic genus *Cryptomaster* Briggs, 1969 was described based on individuals from a single locality in southwestern Oregon. The described species *Cryptomaster leviathan* Briggs, 1969 was named for its large body size compared to most travunioid Laniatores. However, as the generic name suggests, *Cryptomaster* are notoriously difficult to find, and few subsequent collections have been recorded for this genus. Here, we increase sampling of *Cryptomaster* to 15 localities, extending their known range from the Coast Range northeast to the western Cascade Mountains of southern Oregon. Phylogenetic analyses of mitochondrial and nuclear DNA sequence data reveal deep phylogenetic breaks consistent with independently evolving lineages. We use discovery and validation species delimitation approaches to generate and test species hypotheses, including a coalescent species delimitation method to test multi-species hypotheses. For delimited species, we use light microscopy and SEM to discover diagnostic morphological characters. Although *Cryptomaster* has a small geographic distribution, this taxon is consistent with other short-range endemics in having deep phylogenetic breaks indicative of species level divergences. Herein we describe *Cryptomaster behemoth* **sp. n.**, and provide morphological diagnostic characters for identifying *Cryptomaster leviathan* and *Cryptomaster behemoth*.

## Introduction

With more than 4100 described species (Kury 2013), the Opiliones suborder Laniatores is incredibly diverse, and many more species likely await discovery. Despite their diversity, Laniatores is understudied, and many aspects of their phylogeny and evolution remain unknown (Giribet and Sharma 2015). An example is the monotypic genus *Cryptomaster* Briggs, 1969 and its large-bodied (~4 mm) but cryptic species, *Cryptomaster leviathan* Briggs, 1969. *Cryptomaster leviathan* was described from a single locality near the coastal town of Gold Beach, Oregon in the Pacific Northwest (PNW) of the United States. Briggs noted that this new species is remarkably large in size relative to most other Nearctic Laniatores. However, *Cryptomaster leviathan* did not receive further study and remained known only from the type locality until recent published records from the Cascade Range (Derkarabetian et al. 2010) and north of the type locality in the Coast Range (Shear et al. 2014). Given that many Laniatores taxa show high species diversity in small geographic regions (e.g., Ubick and Briggs 1989, Briggs and Ubick 1989, Ubick and Briggs 2002, Derkarabetian and Hedin 2014), these extensions of the known distribution across different mountain ranges indicate the potential for additional species within *Cryptomaster*.

In this study we increase the number of *Cryptomaster* localities to 15, all from mountainous southwestern Oregon. We use multi-locus DNA sequence data to investigate population structure and divergence from samples throughout this range. We recover a deep and concordant phylogenetic split for five loci that is indicative of species level divergence. Discovery and validation species delimitation approaches are used to assess support for multiple species within *Cryptomaster*. Also, we examine morphological differentiation between the divergent genetic groups to provide diagnostic characters for species identification. We delimit two species within *Cryptomaster*, and here describe *Cryptomaster behemoth* sp. n. This research highlights the importance of short-range endemic arachnids for understanding biodiversity (Harvey 2002, Harvey et al. 2011, Keith and Hedin 2012), and further reveals mountainous southern Oregon as a hotspot for endemic animal species (e.g. Shelley 1995, Cokendolpher et al. 2005, Mead et al. 2005, Leonard et al. 2011, Griswold et al. 2012).

## Methods

### Specimen collection

**Figure 1.**
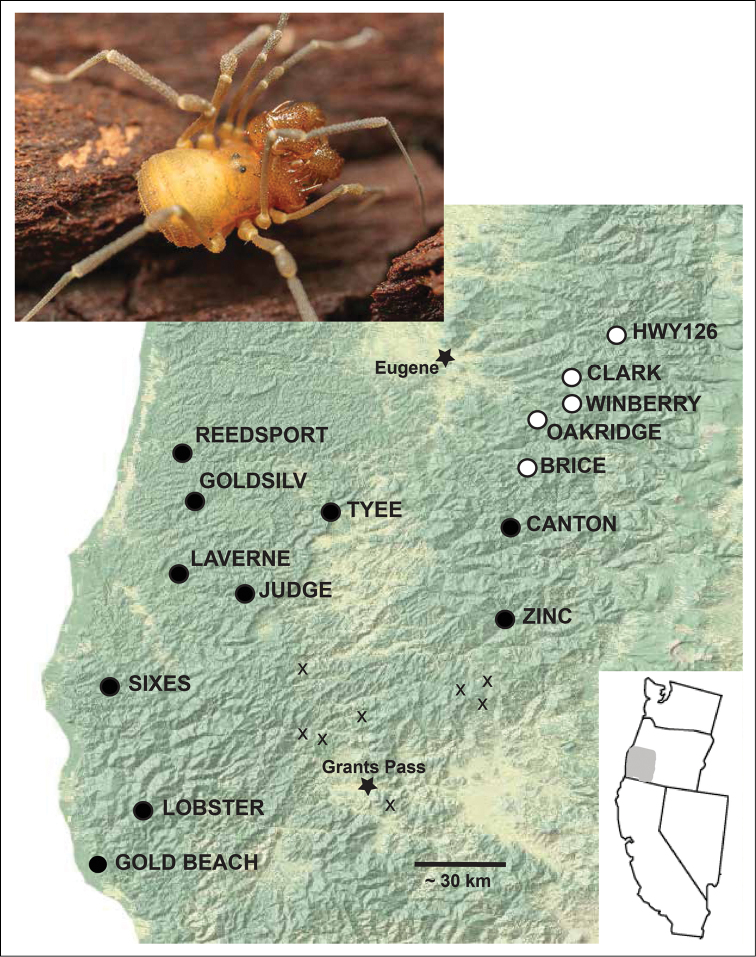
Distribution of *Cryptomaster leviathan* (closed circles) and *Cryptomaster behemoth* (open circles) in southwestern Oregon. Small X’s represent locations in a potential Cascade to Coast corridor where we have collected other travunioids, but not *Cryptomaster*. Inset: male *Cryptomaster leviathan* from the Sixes River location.

We collected 77 *Cryptomaster* individuals from 14 localities in the Coast and Cascade Mountains of southern Oregon (Fig. 1, Suppl. material 1: Table S1), including from near the type locality of *Cryptomaster leviathan* (Gold Beach, OR). *Cryptomaster* are primarily found in mature coniferous forests under woody debris. We attempted collections specifically targeting *Cryptomaster* further north and south of our samples, but these were unsuccessful. Individuals were preserved in 100% EtOH (Koptec) or 80% EtOH for genetic or morphological analysis, respectively. *Speleomaster lexi* Briggs, 1974 was used as an outgroup, following both morphological (Briggs 1974) and molecular evidence (Derkarabetian et al. 2010) that indicate a *Speleomaster* + *Cryptomaster* sister group relationship. Locality data for all specimens are available on the Symbiota Collections of Arthropods Network (http://symbiota4.acis.ufl.edu/scan/portal/index.php). Specimens are housed in the San Diego State Terrestrial Arthropod Collection (SDSU_TAC); type specimens are deposited at the California Academy of Sciences (CASENT9039221).

### Genetic sampling

Genomic DNA was extracted from multiple legs per specimen using the DNeasy Blood and Tissue Kit (Qiagen, Valencia, CA) and the manufacturer’s protocol. Sequence data were obtained for the mitochondrial gene cytochrome c oxidase subunit I (COI), and four nuclear loci (Toll, putative; F-box/LRR-repeat protein, putative; Protein phosphatase 2A regulatory subunit A, putative; Neuromusculin, putative). DNA amplification was performed in a total volume of 25 µL with 1.6 units Platinum Taq (Invitrogen, Carlsbad, CA), 2.2 mM MgCl_2_, 1X PCR buffer, 0.2 mM each dNTP, and 0.4 µM of each primer (Suppl. material 1: Table S2). For COI, cycling conditions consisted of 94 °C for 2 minutes, then 30 cycles of 94 °C for 30 seconds, 50 °C (+ 0.2 °C/cycle) for 40 seconds, and 72 °C for 1.5 minutes, followed by 5 cycles of 94 °C for 30 seconds, 56 °C for 40 seconds, and 72 °C for 1.5 minutes, and then a final 72 °C for 5 minutes. For the nuclear loci, cycling conditions consisted of 94 °C for 3 minutes, then 10 cycles of 94 °C for 1 minute, 63 °C (-0.5 °C/cycle) for 1 minute, and 72 °C for 1 minute, followed by 30 cycles of 94 °C for 15 seconds, 58 °C for 1 minute, and 72 °C for 1 minute, and then a final 72 °C for 5 minutes. PCR products were purified using Montage filter plates and sequenced in both directions by Macrogen USA using the amplification primers. Sequences were edited in Geneious Pro 6 (Kearse et al. 2012) and aligned using MAFFT (Katoh 2013). For heterozygous nuclear sequences, Phase v.2.1.1 (Stephens et al. 2001, Stephens and Scheet 2005) was used to bioinformatically infer alleles.

### Species discovery

Phylogenetic and genetic distance based discovery analyses were used to generate species hypotheses. Maximum likelihood (ML) analyses of individual loci were conducted with RAxML BlackBox v.8.1.11 (Stamatakis et al. 2008) with the GTR + Γ model on the CIPRES web server (Miller et al. 2010), with automatic bootstrap termination. For ML analysis of each locus and *BEAST analyses, the COI dataset was partitioned by codon position, while the less-variable nuclear loci datasets were not partitioned. Genetic diversity statistics were calculated using MEGA v6.06 (Tamura et al. 2013). Automatic Barcode Gap Discovery (ABGD) was applied to the COI data using default settings and the transition/transversion ratio set at 2 (Puillandre et al. 2011). POFAD was run with all nuclear loci using standardized distances calculated with the genpofad option (Joly and Bruneau 2006). POFAD distance results were imported into SplitsTree4 (Huson and Bryant 2006) for reconstruction of a NeighborNet network.

### Species validation

Based on the results of gene tree and species discovery approaches, we compared four alternative species hypotheses using Bayes Factor Delimitation (Grummer et al. 2014). *BEAST v1.8.1 (Drummond et al. 2012) was run with the four nuclear loci under four different species models (see Results, Table 1). Briefly, these species models consisted of: 1) a single species (*Cryptomaster leviathan*), 2) two species, following POFAD results, 3) three species, following the COI gene tree, and 4) four species, following ABGD results. Analyses were run with a strict molecular clock and sequence models determined by jModeltest2 (Guindon and Gascuel 2003, Darriba et al. 2012). Analyses were run for 100,000,000 generations with data stored every 10,000 generations. Log files for all *BEAST runs were visualized in Tracer v.1.6 (Rambaut et al. 2014). Analyses run with GTR sequence models failed to converge, and thus HKY models were applied with the other model parameters as determined by jModeltest2. The Marginal Likelihood Estimate (MLE) was generated based on path sampling (Lartillot and Philippe 2006) and stepping stone (Xie et al. 2011) methods with a chain length of 100,000 generations and pathSteps set at 100. Analyses were run twice and the average MLE was taken for each species model. The Bayes factor was determined by 2*(-ln_HypA_ - -ln_HypB_), with values greater than 10 indicating decisive support for a hypothesis (Kass and Raftery 1995).

**Table 1.** Results of Bayes Factor Delimitation analysis. Species hypotheses are indicated in Fig. 2. Marginal likelihood estimates (MLE) from path sampling (PS) and stepping stone (SS) analyses are shown, with corresponding Bayes Factor (BF) values.

	MLE (PS)	BF	MLE (SS)	BF
4 Species	-4939.87	12.14	-4942.67	13.03
3 Species	-4940.62	13.63	-4943.17	14.03
2 Species	-4933.80	-	-4936.16	-
1 Species	-5010.68	153.75	-5014.01	155.70

### Morphological methods

Linear measurements were taken as in Derkarabetian and Hedin (2014) using an Olympus SZX12 dissecting microscope with an ocular micrometer. For SEM imaging, genitalia were extended from the body by opening the genital and anal opercula, and inserting a small blunt insect pin through the anal operculum and pushing the genitalia out. Specimens were dried using a critical point dryer, mounted on Ted Pella aluminum SEM stubs using copper conductive tape and coated with a 0.6 nm platinum coat. Multiple coats were applied in order to ensure proper coverage and to prevent charging. Coated specimens were imaged on a Quanta 450 SEM at the San Diego State University Electron Microscope Facility. Additionally, genitalia were examined using an Olympus BX40 compound microscope with a drawing tube. Genitalia were dissected from the body as above, then cleared in 10% KOH before viewing.

## Results

### Gene trees and species discovery

**Figure 2.**
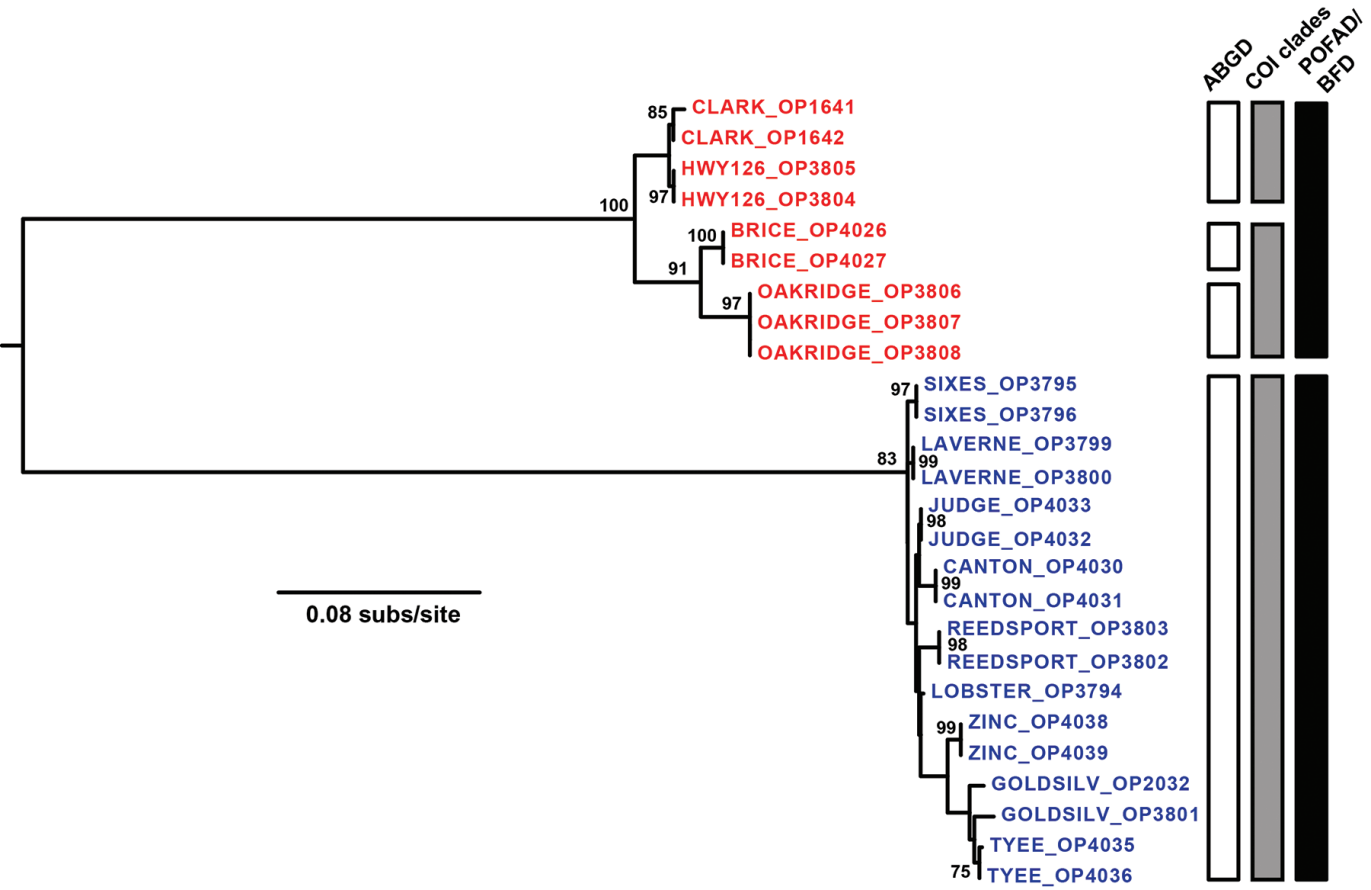
Maximum likelihood COI gene tree with vertical bars showing results of discovery and validation analyses. Blue text = *Cryptomaster leviathan*, red text = *Cryptomaster behemoth*. Numbers adjacent to nodes indicate bootstrap support greater than 70%. The tree was rooted with *Speleomaster lexi* (not shown, see Suppl. material 2: Fig. S1).

**Figure 3.**
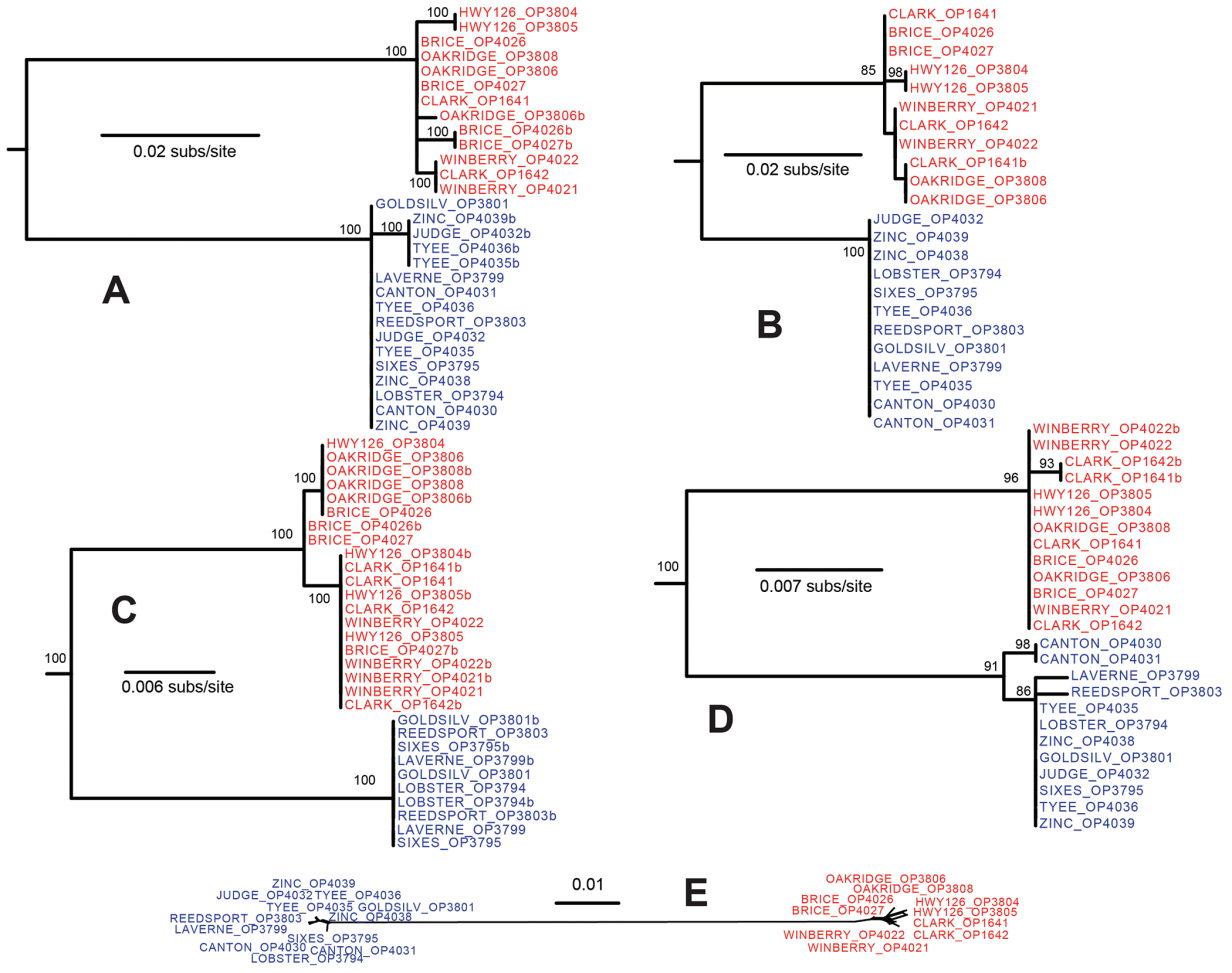
**A–D** Maximum likelihood nuclear gene trees; numbers adjacent to nodes indicate bootstrap support greater than 70%. Trees were rooted with *Speleomaster lexi* (not shown, see Suppl. material 2: Fig. S2–5). Blue text = *Cryptomaster leviathan*, red text = *Cryptomaster behemoth*. **A** Toll **B** F-box/LRR-repeat protein **C** phosphatase 2A protein **D** Neuromusculin **E** NeighborNet network generated from summary distances (ingroup nuclear data only) calculated in POFAD.

Genetic sampling results, GenBank accession numbers, and genetic diversity statistics are summarized in Suppl. material 1: Table S3. Sequence alignments of phased data have been submitted to DRYAD doi: 10.5061/dryad.76rb1. Maximum likelihood analysis of each locus revealed a deep and concordant phylogenetic split within *Cryptomaster* (Figs 2, 3, Suppl. material 2: Figs S1–5). All loci show strong support (bootstrap >83%) for a clade distributed in the Coast Range and southwestern Cascade Range. A second clade with a relatively restricted distribution occurs further northeast in the western Cascade Range (Figs 1–3). Within these clades there is little supported phylogenetic structure with the exception of COI, which further divides the northern Cascade group into a well-supported clade (BS = 91%) consisting of Oakridge+Brice, and a clade with low support (BS = 57%) consisting of Clark+HWY126. The mean p-distance between the two primary clades for COI was 15.5%, and ranged from 3.6-8.2% for the nuclear loci (Suppl. material 1: Table S3).

ABGD analysis of the COI data supported a four species hypothesis (Fig. 2). One species consists of the well-supported COI clade that is distributed in the Coast Range and southwestern Cascades (= *Cryptomaster leviathan*). The remaining three ABGD species occur further north in the Cascade Range and consist of Clark+HWY126, Oakridge, and Brice. POFAD analysis of the nuclear data resulted in two clusters with minimal internal divergence, consistent with the deep phylogenetic split found in all nuclear loci (Fig. 3).

### Species validation

Multiple species hypothesis models were tested using Bayes Factor Delimitation (Fig. 2, Table 1). These hypotheses consisted of 1) a single species of *Cryptomaster*, following Briggs 1969, 2) two species, following POFAD results, 3) three species following the major splits observed in the COI gene tree (*Cryptomaster leviathan*, Clark+HWY126, Oakridge+Brice), and 4) the four species indicated by the ABGD analysis. BF results based on MLE from both path sampling and stepping stone methods (Table 1) indicate decisive support for the two species hypothesis over alternative hypotheses, with a single-species hypothesis most strongly rejected.

### Taxonomy

**Figure 4.**
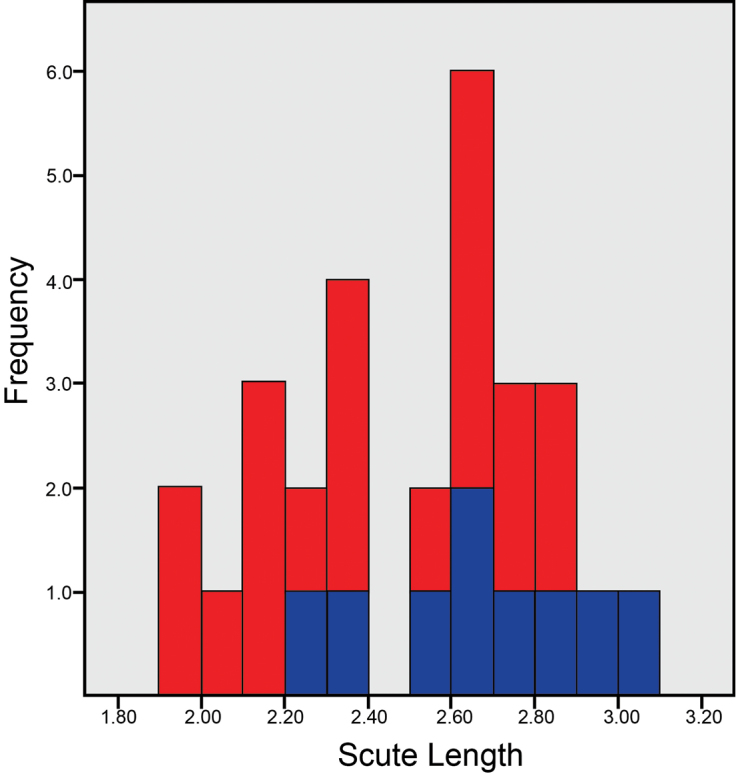
Scute length (mm) distribution of *Cryptomaster*. Figure made using SPSS 22 (IBM Corp. 2013). Blue = *Cryptomaster leviathan*, red = *Cryptomaster behemoth*.

**Figure 5.**
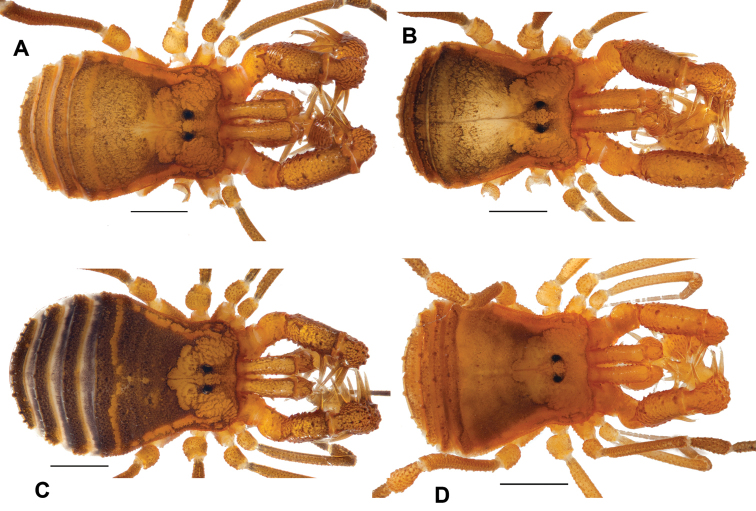
*Cryptomaster* dorsal coloration. **A** Male *Cryptomaster leviathan* (SDSU_OP4039) **B** Holotype male *Cryptomaster behemoth* (CASENT9039221, SDSU_OP4026) **C** Female *Cryptomaster leviathan* (SDSU_OP4037) **D** Allotype female *Cryptomaster behemoth* (CASENT9039221, SDSU_OP4029). Scale bars: 1 mm.

**Figure 6.**
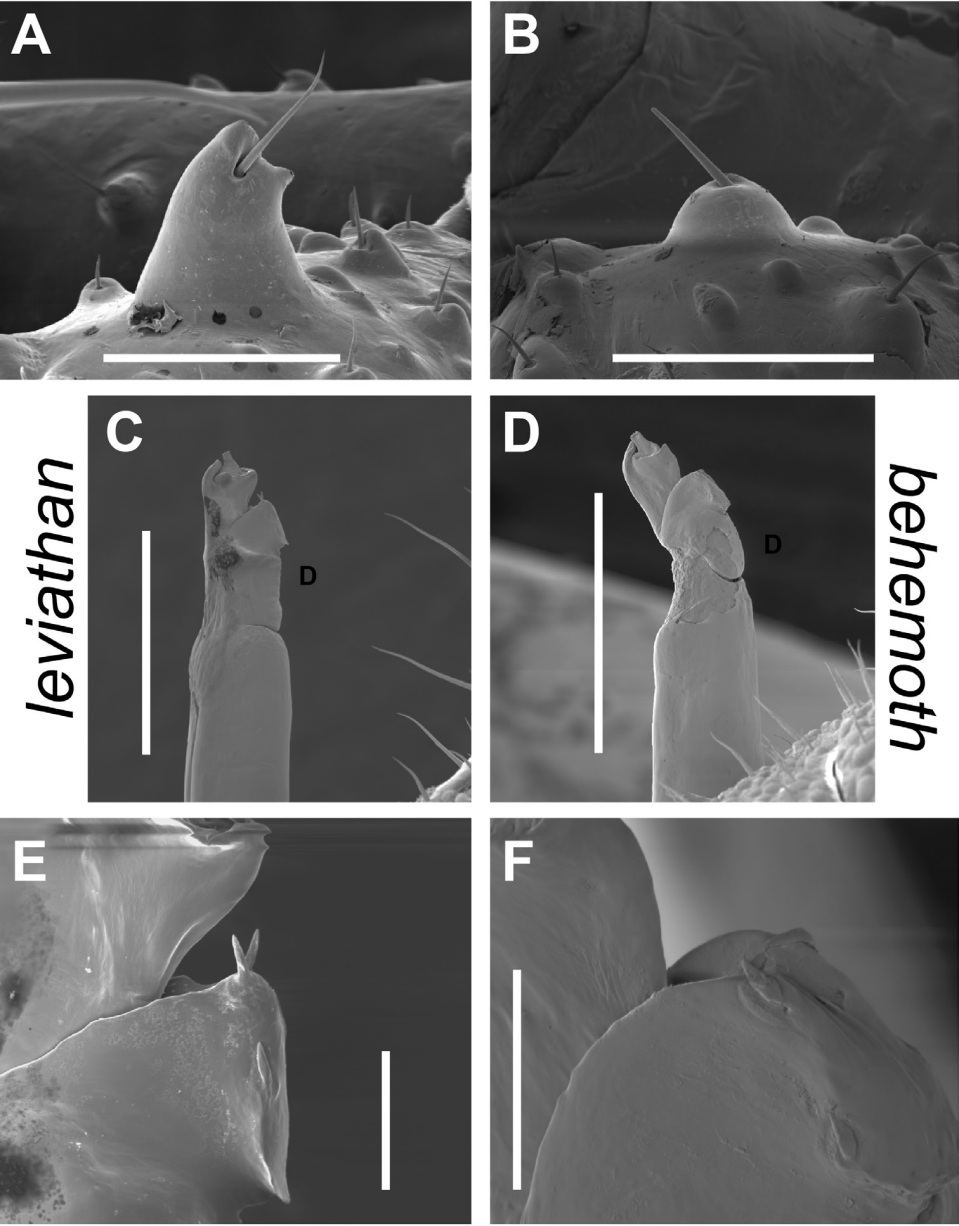
*Cryptomaster* diagnostic morphological characters. **A–B** Lateral view of seta-bearing tubercle found on ventral side of palpal trochanter **A** *Cryptomaster leviathan*, Judge Hamilton **B** *Cryptomaster behemoth*, Brice Creek **C–D** Lateral view of male penis (“D” indicates dorsal side) **C** *Cryptomaster leviathan*, Laverne County Park. **D** *Cryptomaster behemoth*, Oakridge **E–F** Close-up lateral view of penis tip showing spines on dorsal plate **E** *Cryptomaster leviathan*, Laverne County Park, detail showing erect apical spines **F** *Cryptomaster behemoth*, Oakridge, detail showing appressed apical spines. Scale bar: 200 µm (**A, B**); 300 µm (**C–D**); 40 µm (**E–F**).

We note that within each species of *Cryptomaster* two forms are present, a larger and a smaller form, that show a bimodal size distribution (Fig. 4). The basis for these two forms is unknown – the different forms can be found in both sexes, in both species, and from the same localities. Additionally, the two forms are not genetically divergent as COI sequences from different individuals from the same locality are typically most closely related (Fig. 2), and little intraspecific variation exists for the nuclear genes (Fig. 3). Since the original diagnostic characters for the genus *Cryptomaster* (hind claws meeting in 180° opposition, distal swelling on tibia of second leg of males) apply to all examined specimens, we do not redescribe the genus. Here we redescribe *Cryptomaster leviathan* (Fig. 5A, C) from type specimens held at the California Academy of Sciences (CAS) and newly collected material, increasing our understanding of *Cryptomaster leviathan* with new localities (Fig. 1, Suppl. material 1: Table S1) and morphological data (Suppl. material 1: Table S4) from across its range. Additionally, we describe the new species *Cryptomaster behemoth* sp. n. (Fig. 5B, D), providing diagnostic characters for both species (Fig. 6).

Morphological abbreviations: DCS = distal cheliceral segment , GO = genital operculum , LII = leg II , OC = ocularium , PCS = proximal cheliceral segment , PF = pedipalpal femur , PT = pedipalpal trochanter , SBT = seta-bearing tubercle . All measurements are in millimeters. Morphological images have been submitted to Morphbank.

#### Cryptomaster leviathan

Taxon classificationAnimaliaOpilionesCladonychiidae

Briggs, 1969

Figures: map 1 ; habitus 5A, C , 7A, C, E ; somatic 6A , 8A, C, E ; penis 6C, E , 9A, C, E ; ovipositor 10A–E

Cryptomaster leviathan Briggs, 1969: 41–43, figures 15–25.

##### Type material examined.

**Holotype** male and five female paratypes from 4.5 miles south of Gold Beach, Curry County, Oregon, 29 January 1967, under spruce bark in virgin spruce forest, coll. T. Briggs, V. Lee, and K. Hom.

##### Diagnosis.

This species differs from *Cryptomaster behemoth* in having the enlarged SBT of PT acute (Fig. 6A), and keel-shaped protrusion of dorsal plate of penis with apical pair of spines fully erect and directed along the longitudinal axis of the penis (Fig. 6C, E).

##### Genetic data.

GenBank Accession numbers: KU059639-KU059655, KU059667-KU059678, KU059690-KU059701, KU059713-KU059717, KU059729-KU059740.

##### Morphbank images.

SDSU_TAC000021, Morphbank Specimen ID: 855927

<http://www.morphbank.net/?id=855927>, 14 SEM images

SDSU_TAC000022, Morphbank Specimen ID: 855928

<http://www.morphbank.net/?id=855928>, 7 SEM images

SDSU_TAC000027, Morphbank Specimen ID: 855931

<http://www.morphbank.net/?id=855931>, 17 SEM images

SDSU_TAC000204, Morphbank Specimen ID: 855933

<http://www.morphbank.net/?id=855933>, 19 SEM images

SDSU_TAC000248, Morphbank Specimen ID: 856245

<http://www.morphbank.net/?id=856245>, 4 SEM images

##### Redescription.

MALE: Measurements of holotype male, with the average and range of all three specimens measured in parentheses (Suppl. material 1: Table S4).

Body length 3.44, scute length 2.75 (2.71; 2.5–2.89), scute width 3.06 (2.75; 2.5–3.06), prosoma width 2.05 (1.95; 1.81–2.05). Shoulder tubercles present but small. Scute microgranulate. Holotype discolored due to preservation; for other specimens, integument color contrasts dorsally at midline between prosoma and opisthosoma, although not as strongly as in females. OC width 0.59 (0.55; 0.49–0.59). Ventral surface microgranulate. GO missing in holotype, length 0.3–0.34, width 0.28–0.31.

PT with acute mesal SBT. PF length 2.01 (1.98; 1.8–2.13), PF depth 0.72 (0.69; 0.64–0.72), with 7 (sometimes 5–6) spines, with the basal pair prominent, 3 (sometimes 4) enlarged dorsal spines, and 2 enlarged prolateral spines distally. PCS with 3 anterior spines dorsally and with 2 or 3 small retrolateral spines; DCS with 2 rows of small, dorsal, forward-facing acute SBTs. PCS width 0.42, DCS length 1.6, DCS width 0.46.

Trochanter 0.57, femur 0.92, patella 0.7, tibia 1.48, metatarsus 1.72, tarsus 1.04. LII length 11.19 (11.31; 10.69–12.06): trochanter 0.62 (0.6; 0.58–0.62), femur 2.73 (2.73; 2.55–2.91), patella 0.94 (0.89; 0.77–0.95), tibia 2.3 (2.37; 2.28–2.55), metatarsus 2.49 (2.61; 2.49–2.8); tarsus 2.1 (2.11; 1.99–2.25); tibia distally and ventrally swollen, with 5 rounded SBTs, 1–3 with setae twisted. Tarsal count 5-[11–15]-5–6.

Penis elongate; glans laterally compressed, dorsal plate extending outward into a more angled and acute keel shaped protrusion, with two pairs of spines, apical pair erect (pointing along the longitudinal axis of the penis), subapical pair appressed to dorsal plate; ventral plate cultriform with dorsally curved apical process.

FEMALE: Nineteen total individuals examined, including five paratypes; average measurements taken for subset (Suppl. material 1: Table S4), with range of all seven specimens measured in parentheses. Descriptive characters as for males unless otherwise noted.

Scute length 2.82 (2.69–3.03), scute width 3.01 (2.84–3.13), prosoma width 1.96 (1.8–2.14). Relative to males very dark, with strong contrast at midline between light-brown prosoma and dark-brown opisthosoma. OC width 0.56 (0.51–0.6). GO length 0.37 (0.33–0.39), width 0.38 (0.34–0.4).

PT mesal SBT acute. PF length 1.84 (1.68–1.94), PF depth 0.63 (0.58–0.67), usually with 5 (up to 7) ventral spines, 4 dorsal spines (2 to 6), and 2 distal prolateral spines. PCS with 2–3 anterior spines dorsally, with 1–4 small retrolateral spines.

LII length 10.23 (9.61–10.81): trochanter 0.58 (0.54–0.67), femur 2.53 (2.35–2.63), patella 0.83 (0.76–0.89), tibia 2.16 (1.99–2.29), metatarsus 2.39 (2.16–2.55), tarsus 1.92 (1.81–2.0); tibia without distal ventral swelling.

Ovipositor with four lobes, lateral lobes largest with seven apical setae, and a single large spine with a bifurcate tip, ventral lobe smallest.

**Figure 7.**
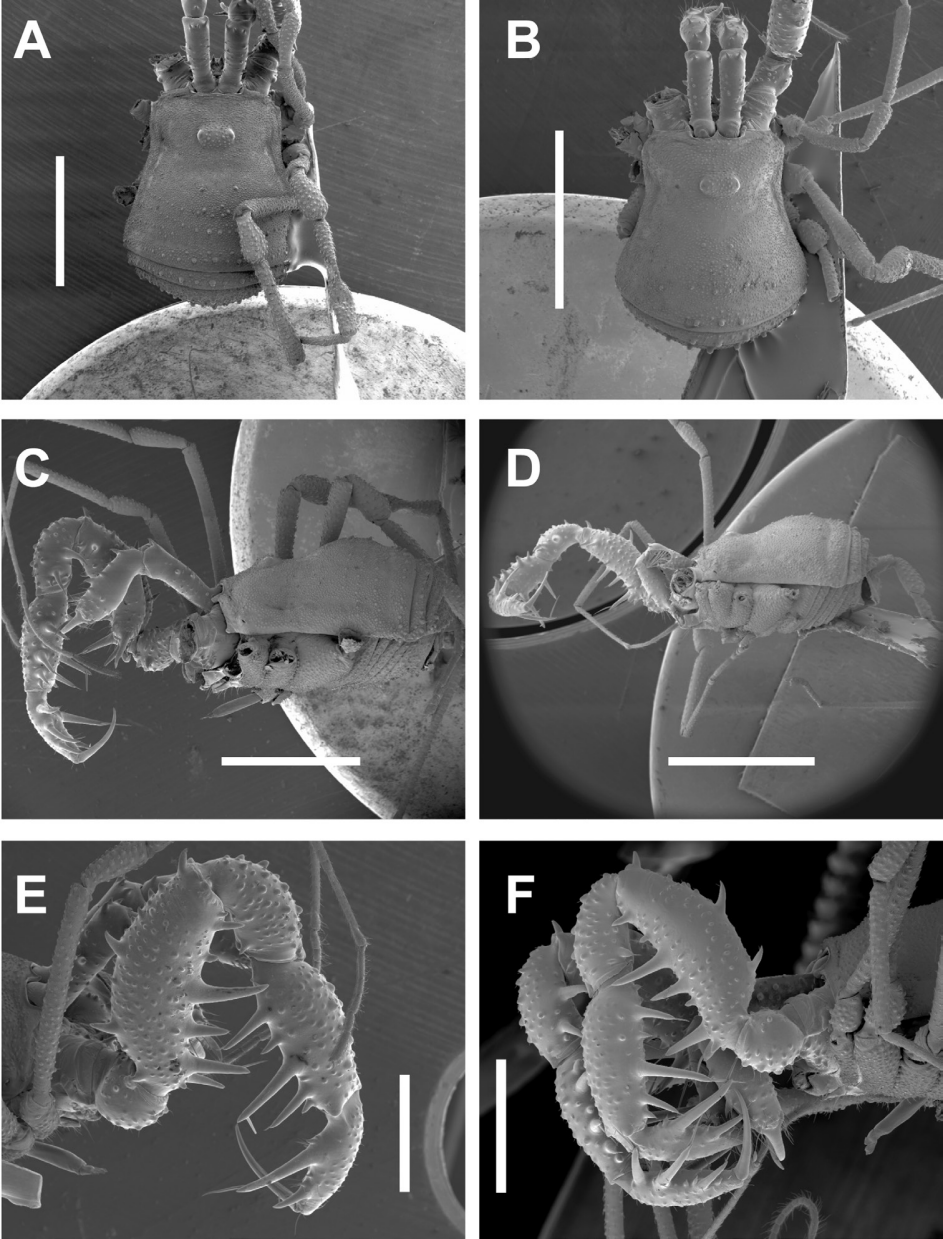
*Cryptomaster* habitus. **A–B** Habitus, dorsal **A** *Cryptomaster leviathan*, Judge Hamilton **B** *Cryptomaster behemoth*, Brice Creek **C–D** Habitus, lateral **C** *Cryptomaster leviathan*, Judge Hamilton **D** *Cryptomaster behemoth*, Oakridge (female) **E–F** Pedipalp, retrolateral **E** *Cryptomaster leviathan*, Judge Hamilton **F** *Cryptomaster behemoth*, Oakridge. Scale bar: 2 mm (**A, B, C, D**); 1 mm (**E–F**).

**Figure 8.**
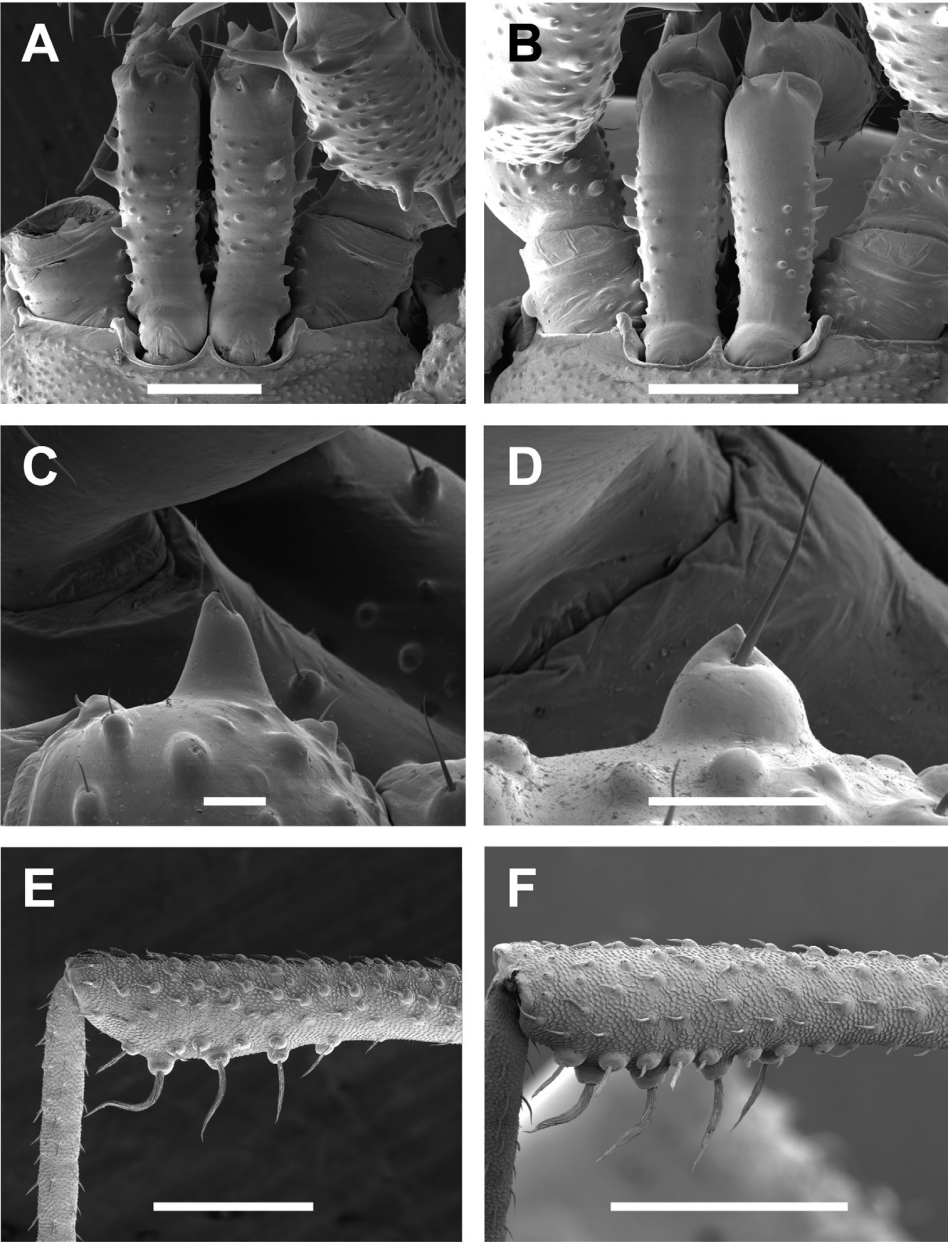
*Cryptomaster* appendages. **A–B** Proximal cheliceral segment, dorsal **A** *Cryptomaster leviathan*, Reedsport **B** *Cryptomaster behemoth*, Oakridge **C–D** SBT of palpal trochanter, lateral **C** *Cryptomaster leviathan*, Laverne County Park **D** *Cryptomaster behemoth*, Oakridge **E–F** Leg II tibia, distal swelling, retrolateral **E** *Cryptomaster leviathan*, Judge Hamilton **F** *Cryptomaster behemoth*, Oakridge. Scale bar: 500 µm (**A, B**); 100 µm (**C, D**); 400 µm (**E, F**).

**Figure 9.**
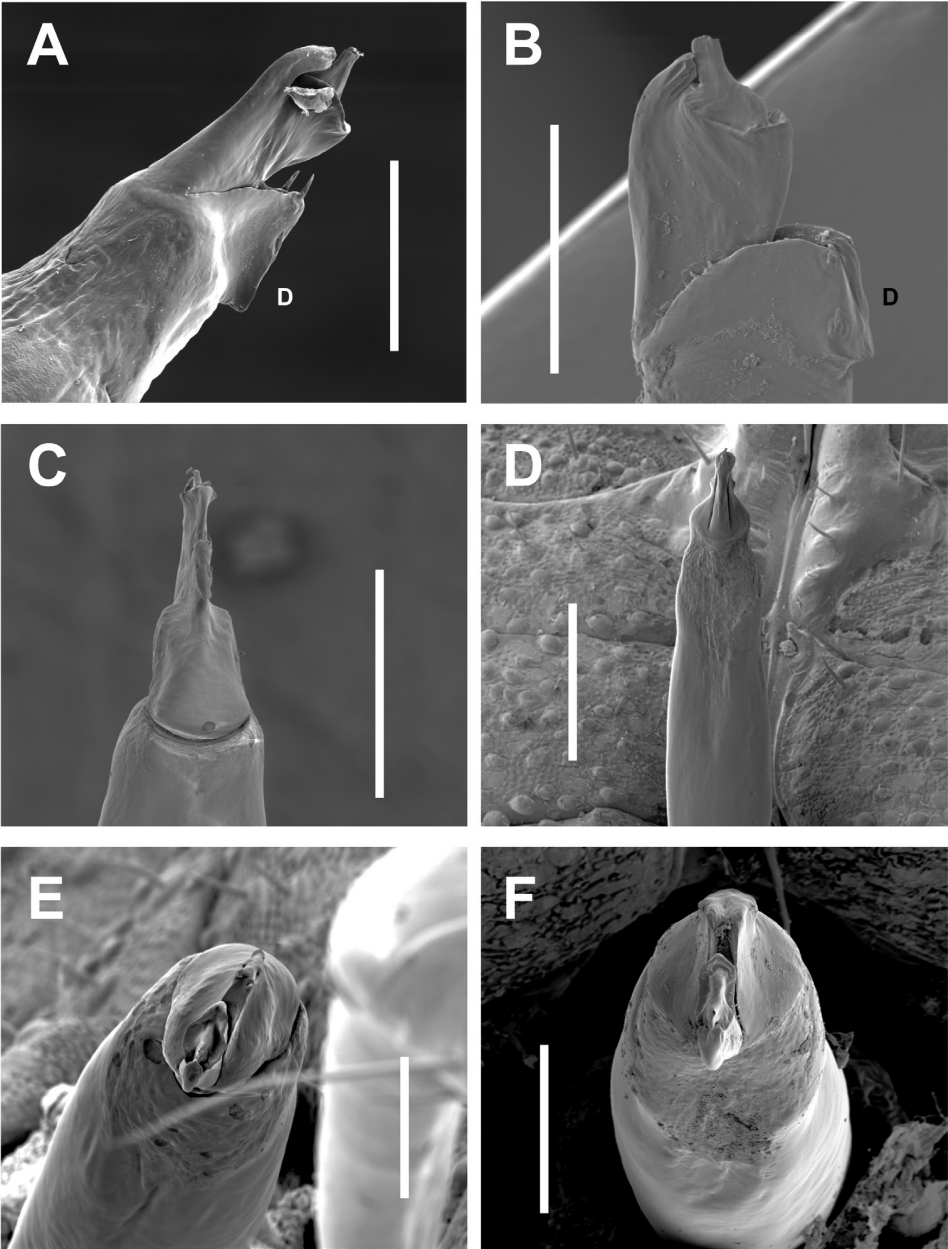
*Cryptomaster* penises. **A–B** Apicolateral (“D” indicates dorsal side) **A** *Cryptomaster leviathan*, Laverne County Park **B** *Cryptomaster behemoth*, Brice Creek **C–D** Apicodorsal **C** *Cryptomaster leviathan*, Judge Hamilton **D** *Cryptomaster behemoth*, Oakridge **E–F** Apical **E** *Cryptomaster leviathan*, Judge Hamilton **F** *Cryptomaster behemoth*, Brice Creek. Scale bar: 100 µm (**A, B, E, F**); 200 µm (**C, D**).

**Figure 10.**
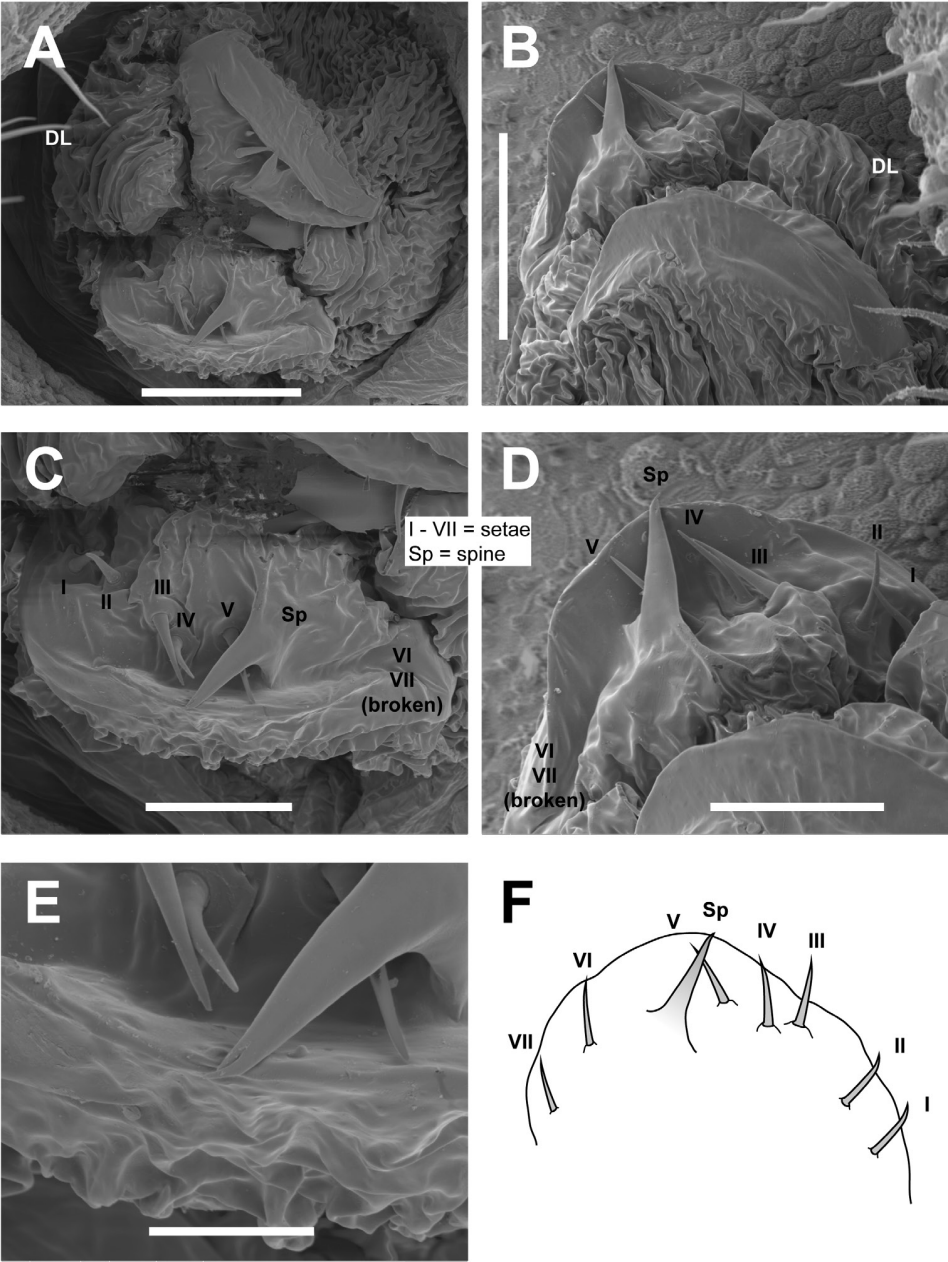
*Cryptomaster* ovipositors. **A–E** *Cryptomaster leviathan*, Reedsport **A** Apical (“DL” indicates dorsal lobe) **B** Lateral **C** Apical, emphasizing setae and spine arrangement **D** Lateral, emphasizing setae and spine arrangement **E** Apical, spine **F** *Cryptomaster behemoth*, Oakridge, sagittal view, emphasizing setae and spine arrangement, 40×. Scale bar: 100 µm (**A, B**); 50 µm (**C, D**); 25 µm (**E**).

##### Other material examined.

See Suppl. material 1: Table S1 and S4 for locality information of all specimens examined.

##### Distribution and habitat.

For specific localities, habitats, and microhabitats see Suppl. material 1: Table S1. This species is distributed in southwestern Oregon including throughout the southern Oregon Coast Range from the Umpqua River to the Coquille River, and south into the Klamath Mountains to the Rogue River. The range extends east to the southern Oregon Cascade Mountains in the South Umpqua and North Umpqua River Basins. *Cryptomaster leviathan* is typically associated with mature coniferous or mixed coniferous and hardwood forests, but has also been found in disturbed forests and forests with few conifers. Specimens are most often found under large woody debris associated with decaying logs and stumps, and in *Acer* and *Polystichum* leaf litter.

#### Cryptomaster behemoth

Taxon classificationAnimaliaOpilionesCladonychiidae

Starrett & Derkarabetian sp. n.

http://zoobank.org/6F8DCC84-A59D-4FEC-AB7C-0A05678D8223

Figures: map 1 ; habitus 5B, D , 7B, D, F ; somatic 6B , 8B, D, F ; penis 6D, F , 9B, D, F ; ovipositor 10F

Cryptomaster leviathan [partim] Derkarabetian et al. (2010)
Cryptomaster leviathan [partim] Shear et al. (2014)

##### Etymology.

The specific epithet is a noun in apposition, which refers to the large size of this species. Like *leviathan*, the specific epithet *behemoth* is derived from Hebrew; these are the names of two large and powerful beasts mentioned in the Book of Job.

##### Type material.

**Holotype** male and allotype female (deposited in CAS, CASENT9039221; SDSU_OP4026, SDSU_OP4029) from near Brice Creek, Brice Creek Road, 3.3 miles southeast of Forest Service Road 17, Umpqua National Forest, Lane County, Oregon; N43.6749°, W122.7290°; elevation 418 m; 29 March 2015; habitat: *Acer macrophyllum*, *Thuja plicata*, *Pseudotsuga menziesii*, *Polystichum munitum* forest; in and under large woody debris and other forest litter; collectors: J. Starrett, S. Derkarabetian, A. Cabrero, C. Richart. **Paratypes**: One female (deposited in CAS) from identical locality and information as holotype and allotype. Three females (two deposited in CAS, 1 deposited in SDSU_TAC; SDSU_TAC0000023) from Goodman Creek Road, off OR 58, northwest of Oakridge, Lane County, Oregon; N43.8429°, W122.6854°; elevation 340 m; 19 August 2014; habitat: old growth Douglas fir forest/woody debris; collectors: M Hedin, E Ciaccio, A Cabrero, J Starrett, S Derkarabetian. Two females (one each deposited in CAS and SDSU_TAC; SDSU_TAC0000234) from Brice Creek, Brice Creek Road, 3.3 miles southeast of FS 17, Umpqua National Forest, Lane County, Oregon; N43.6760°, W122.7290°; elevation 418 m; 29 March 2015; habitat: *Acer macrophyllum*, *Thuja plicata*, *Pseudotsuga menziesii*, *Polystichum munitum* forest; woody debris and litter; collectors: J Starrett, S Derkarabetian, A Cabrero, C Richart. One female (deposited in SDSU_TAC; SDSU_TAC0000028) from Highway 126, near Quartz Creek Road, Lane County, Oregon; N44.1248°, W122.3846°; elevation 300 m; 19 August 2014; habitat: decent *Pseudotsuga menziesii* forest; woody debris; collectors: M Hedin, E Ciaccio, A Cabrero, J Starrett, S Derkarabetian.

##### Diagnosis.

This species differs from *Cryptomaster leviathan* by having the enlarged SBT of PT rounded (Fig. 6B), and keel-shaped protrusion of dorsal plate of penis with apical pair of spines appressed and perpendicular to the longitudinal axis of the penis (Fig. 6D, F).

##### Genetic data.

GenBank Accession numbers: HM056724, KU059631-KU59638, KU059657-KU059666, KU059680-KU059689, KU059703-KU059712, KU059719-KU59728.

##### Morphbank images.

CASENT9039221, Holotype, Morphbank Specimen ID: 855951

<http://www.morphbank.net/?id=855951>, 1 image

CASENT9039221, Paratype, Morphbank Specimen ID 855929,

<http://www.morphbank.net/?id=855929>, 1 image

SDSU_TAC000023.5, Morphbank Specimen ID: 855929

<http://www.morphbank.net/?id=855929>, 1 SEM image

SDSU_TAC000023.6, Morphbank Specimen ID: 855930

<http://www.morphbank.net/?id=855930>, 12 SEM images

SDSU_TAC000203, Morphbank Specimen ID: 855932

<http://www.morphbank.net/?id=855932>, 16 SEM images

SDSU_OP1641, GUID: 38c9a86e-088d-4040-8988-af37fa74ad84

<http://symbiota4.acis.ufl.edu/scan/portal/collections/individual/index.php?occid=14702249>, 1 image

SDSU_OP1641, Morphbank Specimen ID: 835725

<http://www.morphbank.net/?id=835725>, 2 images

SDSU_OP1642, GUID: 8558ef80-a8c7-439d-bd93-dba8ec8d11d4

<http://symbiota4.acis.ufl.edu/scan/portal/collections/individual/index.php?occid=14702250>, 1 image

##### Description.

MALE: Measurements of holotype male, with the average and range of all nine specimens measured in parentheses (Suppl. material 1: Table S4).

Body length 3.40, scute length 2.69 (2.46; 1.97–2.75), scute width 2.58 (2.41; 1.97–2.75), prosoma width 1.88 (1.77; 1.48–1.94). Shoulder tubercles present but small. Scute microgranulate. Integument color without contrast dorsally at midline between prosoma and opisthosoma in all individuals. OC a low broad mound; height 0.14; width 0.59 (0.52; 0.39–0.59). Eye color dark brown; surrounding integument with black pigment. Ventral surface microgranulate. GO length 0.32 (0.31; 0.27–0.32), width 0.28 (0.27; 0.26–0.29).

Mesal SBT of PT relatively low, rounded, with seta near apex of tubercle. PF length 2.0 (1.78; 1.38–2.03), PF depth 0.76 (0.65; 0.44–0.8), with 4–6 ventral spines, with the basal pair prominent, usually 3 enlarged dorsal spines (sometimes 4), and 2 enlarged distal prolateral spines. Pedipalp patella with 2 (one specimen with 3) enlarged prolateral spines and 1 ventroretrolateral spine; tibia with rows of 5 enlarged pro- and retrolateral spines; tarsus with 3 prolateral and 2 retrolateral enlarged spines. PCS with 2 dorsal anterior spines (sometimes 1–3); and with 2 small retrolateral spines (sometimes 1); DCS with 2 rows of small, dorsal, forward-facing acute SBTs. PCS width 0.37, DCS length 1.63, DCS width 0.47.

Leg II length 10.59 (9.88; 8.15–11.0); trochanter 0.58 (0.53; 0.41–0.59), femur 2.66 (2.45; 1.99–2.72), patella 0.84 (0.8; 0.64–0.89), tibia 2.26 (2.14; 1.73–2.4), metatarsus 2.33 (2.22; 1.82–2.48), tarsus 1.93 (1.81; 1.54–1.97); tibia distally and ventrally swollen, with 3–5 rounded SBTs, 2–4 with setae twisted. Tarsal claw as for genus. Tarsal count 5–13–5–6; variation exists in the number of LII tarsal segments.

Penis elongate; glans laterally compressed, dorsal plate extending outward into a more rounded keel shaped protrusion, with two pairs of spines, both pairs appressed to plate (perpendicular to the longitudinal axis of the penis); ventral plate cultriform with dorsally curved apical process.

FEMALE: Measurements of allotype female, with the average and range of all 10 specimens measured in parentheses (Suppl. material 1: Table S4). Descriptive characters as for males unless otherwise noted.

Body length 2.94, scute length 2.35 (2.29; 2.05–2.69), scute width 2.5 (2.52; 2.23–2.75), prosoma width 1.62 (1.61; 1.49–1.76). Integument color darker than males, usually with light contrast dorsally at midline between prosoma and opisthosoma (in 7 of 8 individuals). OC height 0.12; width 0.47 (0.47; 0.4–0.52).

Mesal SBT of PT relatively low, rounded, with seta near apex of tubercle. PF length 1.53 (1.48; 1.39–1.64), PF depth 0.54 (0.53; 0.47–0.61), with 6 or 7 ventral spines (sometimes 5), with the basal pair prominent, with 3 enlarged dorsal spines (sometimes 4), and 2 enlarged distal prolateral spines (sometimes 1). Pedipalp patella with 2 enlarged prolateral spines and 1 ventroretrolateral spine. PCS with 2 anterior spines dorsally, with 2 small retrolateral spines; PCS length 0.30; DCS length 1.19, width 0.28.

LII length 8.66 (8.51; 8.15–9.38); trochanter 0.5 (0.49; 0.44–0.59), femur 2.15 (2.06; 1.95–2.29), patella 0.68 (0.69; 0.66–0.76), tibia 1.78 (1.79; 1.68–1.96), metatarsus 1.95 (1.91; 1.82–2.1), tarsus 1.6 (1.57; 1.55–1.69); Tarsal count 5–11–5-6; variation exists in the number of LII tarsal segments.

GO length 0.3 (0.3; 0.27–0.34), width 0.29 (0.3; 0.27–0.36).

Ovipositor with four lobes, lateral lobes largest with seven apical setae, and a single large spine with a bifurcate tip, ventral lobe smallest.

##### Other material examined.

See Suppl. material 1: Tables S1 and S4 for locality information of all specimens examined.

##### Distribution and Habitat.

For specific localities, habitats, and microhabitats see Suppl. material 1: Table S1. This species is distributed in the central Cascade Mountains of Oregon east and southeast of Eugene from Brice Creek in the Row River Drainage north to the north side of the McKenzie, with all known localities in Lane County. It is possible that populations occur further north in the western Cascades (Fig. 1). Habitats and microhabitats do not obviously differ from *Cryptomaster leviathan*, found in mature coniferous or mixed coniferous and hardwood forests, most often associated with large woody debris and bark.

## Discussion

### Species delimitation and short range endemic taxa

*Cryptomaster* exhibits a deep molecular phylogenetic break consistent with species level divergence, similar to that observed in many other harvestmen taxa (Boyer et al. 2007a, Thomas and Hedin 2008, Hedin and Thomas 2010, Schönhofer and Martens 2010, Derkarabetian et al. 2011, Richart and Hedin 2013, Fernández and Giribet 2014). Based on analyses of genetic data using discovery and validation approaches, we conclude that *Cryptomaster* consists of two species. We found complete genealogical concordance in the mitochondrial and nuclear loci sampled, indicating strong evidence for long-term reproductive isolation between *Cryptomaster leviathan* and *Cryptomaster behemoth* (Avise and Ball 1990). While gene tree and ABGD analyses of COI data indicate additional potential species, mitochondrial datasets are known to over-split short range endemic arachnid taxa (e.g., Keith and Hedin 2012, Satler et al. 2013, Derkarabetian and Hedin 2014, Fernández and Giribet 2014, Hamilton et al. 2014, Leavitt et al. 2015, Hedin et al. 2015, Wachter et al. 2015). Thus, we favor the more conservative two-species hypothesis, which is supported by the multi-species coalescent validation approach using four independent nuclear loci.

Our sampling efforts greatly increased the known range of *Cryptomaster*, yet both species still appear to have limited distributions. Interestingly, the range for *Cryptomaster leviathan* extends across multiple mountain ranges, yet little to no genetic structure exists between these ranges. Conversely, *Cryptomaster behemoth* has a particularly small range, yet harbors higher population genetic structure. This could be due to the greater topographic complexity of the central Cascade Range, or these populations may have persisted in their current locations for a longer time compared to *Cryptomaster leviathan* populations. A pattern of deep population structure in topographically complex regions is consistent with numerous other arachnid taxa (Hendrixson and Bond 2005, Boyer et al. 2007a, Thomas and Hedin 2008, Bryson et al. 2013, Hedin et al. 2013, Esposito et al. 2015).

### Biogeographic Uncertainty

Short-range endemic taxa have been shown to help elucidate ancient biogeographic processes (Boyer et al. 2007b, Bryson et al. 2013, Hedin et al. 2013). However, given the deep genetic break between *Cryptomaster leviathan* and *Cryptomaster behemoth*, contrasting with minimal population structure within these taxa, it is difficult to decipher the processes that led to the division of these species. *Cryptomaster leviathan* is comparatively much more widespread with populations in the Klamath Mountains, southern Oregon Coast Ranges, and the Cascade Mountains, although these ranges appear to be connected by corridors of possibly suitable habitat (Fig. 1). Speciation may have occurred while *Cryptomaster leviathan* and *Cryptomaster behemoth* inhabited separate mountain ranges and *Cryptomaster leviathan* later dispersed from the coast northeast to the Cascade Range. Alternatively, the two species may have diverged within the Cascade Range. Under this scenario, *Cryptomaster leviathan* could have already been present in the Coast Range or dispersed west from the Cascades subsequent to speciation. These species currently occupy different river drainage systems, separated by the relatively high-elevation Calapooya Divide, but it remains unclear whether this represents a primary or secondary barrier to dispersal. Sampling of faster evolving loci and additional fine-scale geographic sampling is needed to test these alternative hypotheses.

## Supplementary Material

XML Treatment for Cryptomaster leviathan

XML Treatment for Cryptomaster behemoth

## Supplementary materials

Supplementary material 1 Supplementary Table 1–4 Data type: Excel Table Explanation note: Table S1. Locality data Table S2. PCR primer information Table S3. GenBank accession information and genetic diversity statistics Table S4. Morphological measurements This dataset is made available under the Open Database License (http://opendatacommons.org/licenses/odbl/1.0/). The Open Database License (ODbL) is a license agreement intended to allow users to freely share, modify, and use this Dataset while maintaining this same freedom for others, provided that the original source and author(s) are credited.James Starrett, Shahan Derkarabetian, Casey H. Richart, Allan Cabrero, Marshal Hedin

Supplementary material 2 Supplementary Figures 1–5 Data type: figures Explanation note: Figure S1. Maximum likelihood gene tree for cytochrome c oxidase 1 (COI) locus. Numbers adjacent to nodes indicate bootstrap support greater than 70%. Figure S2. Maximum likelihood gene tree for Toll locus. Numbers adjacent to nodes indicate bootstrap support greater than 70%. Figure S3. Maximum likelihood gene tree for F-box/LRR-repeat locus. Numbers adjacent to nodes indicate bootstrap support greater than 70%. Figure S4. Maximum likelihood gene tree for phosphatase 2A locus. Numbers adjacent to nodes indicate bootstrap support greater than 70%. Figure S5. Maximum likelihood gene tree for Neuromusculin locus. Numbers adjacent to nodes indicate bootstrap support greater than 70%. This dataset is made available under the Open Database License (http://opendatacommons.org/licenses/odbl/1.0/). The Open Database License (ODbL) is a license agreement intended to allow users to freely share, modify, and use this Dataset while maintaining this same freedom for others, provided that the original source and author(s) are credited.James Starrett, Shahan Derkarabetian, Casey H. Richart, Allan Cabrero, Marshal Hedin
