# Supplementary material for: A new monster from southwest Oregon forests: Cryptomaster behemoth sp. n. (Opiliones, Laniatores, Travunioidea)
Source: Zookeys. 2016 Jan 20;(555):11–35. doi: 10.3897/zookeys.555.6274 (PMC4740820; doi:10.3897/zookeys.555.6274)
Supplement: Supplementary material 2 — Supplementary Figures 1–5 [file zookeys-555-011-s002.pdf]

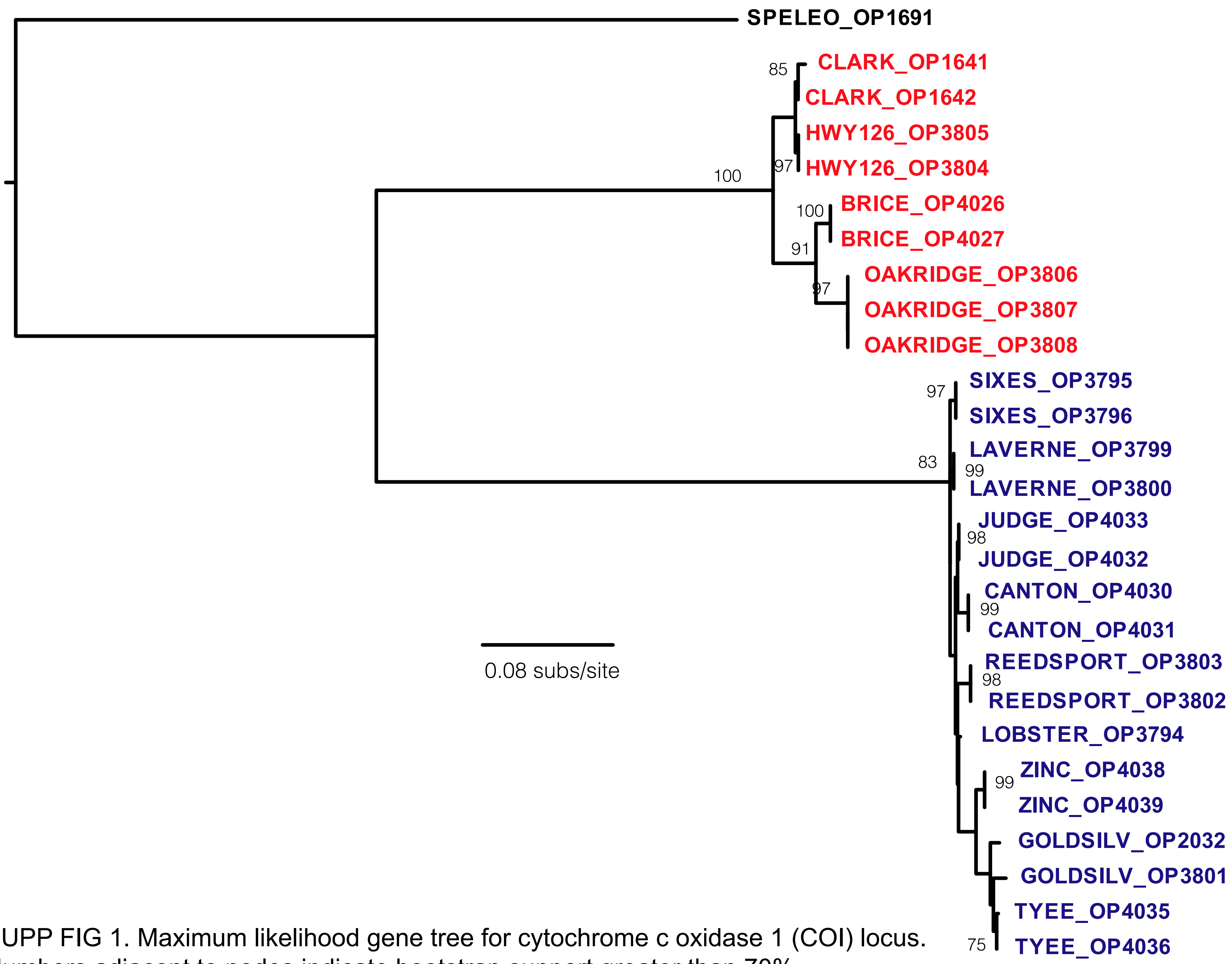

SUPP FIG 1. Maximum likelihood gene tree for cytochrome c oxidase 1 (COI) locus. Numbers adjacent to nodes indicate bootstrap support greater than 70%.

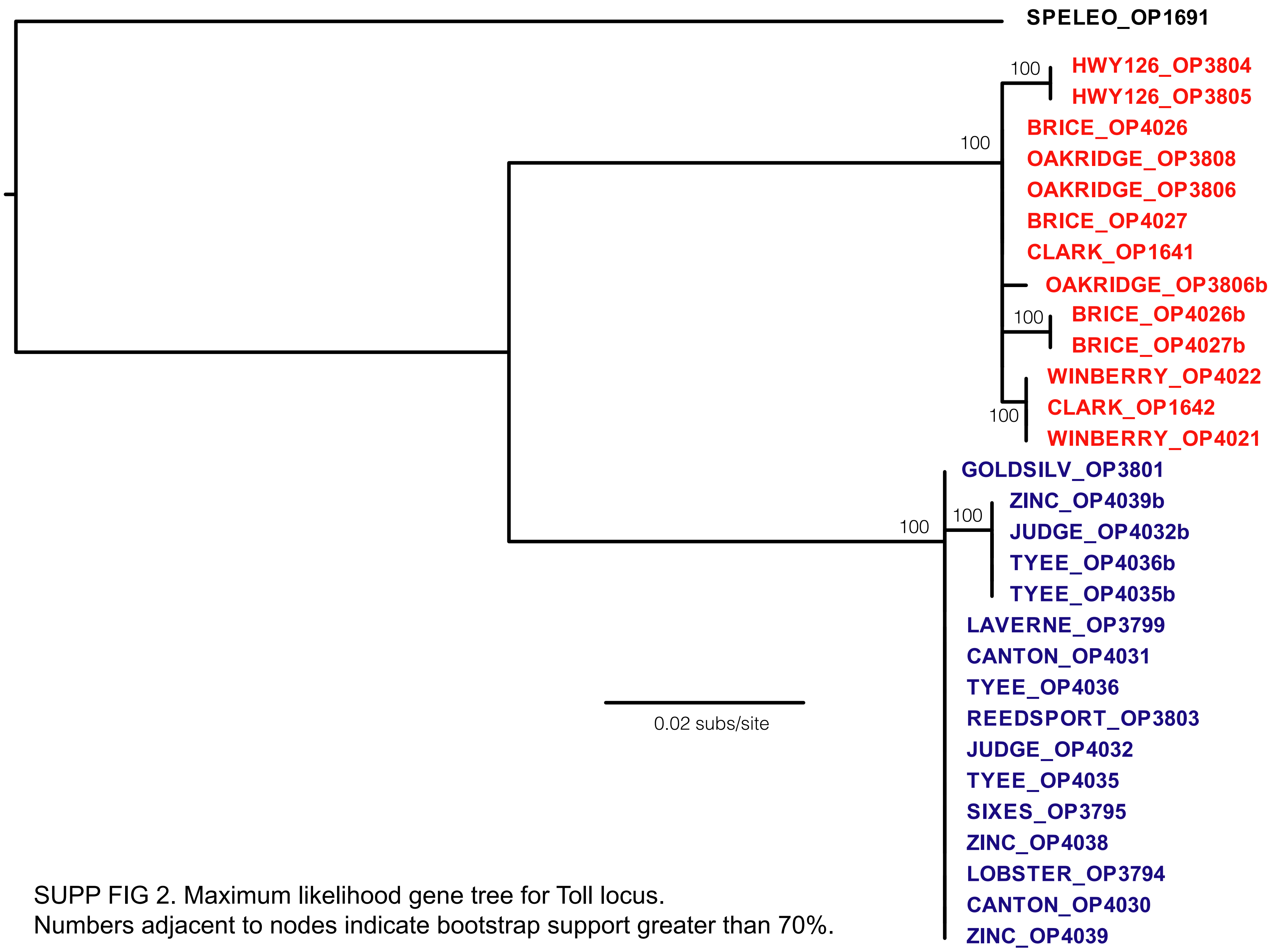

SUPP FIG 2. Maximum likelihood gene tree for Toll locus.
Numbers adjacent to nodes indicate bootstrap support greater than 70%.

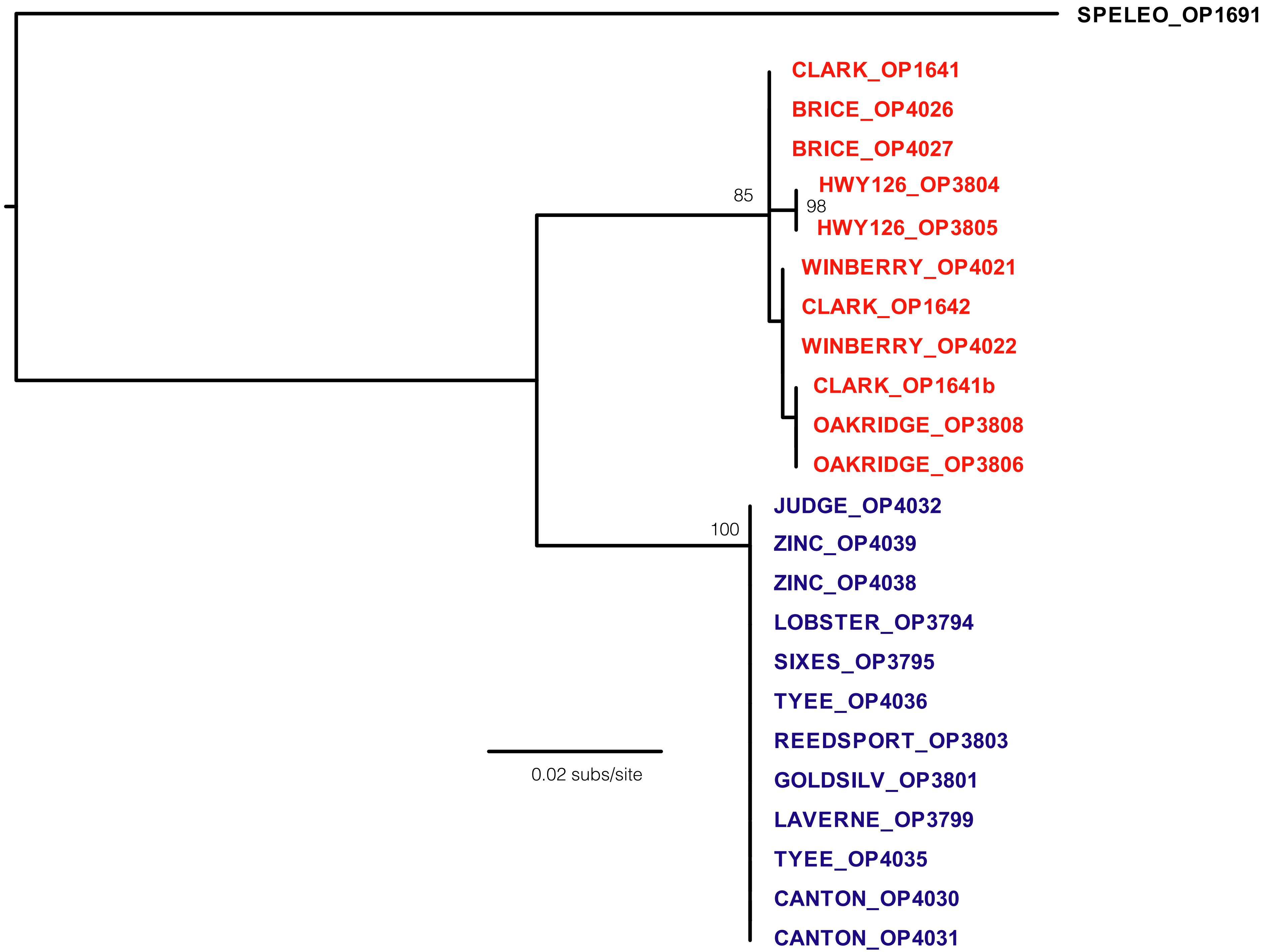

SUPP FIG 3. Maximum likelihood gene tree for F-box/LRR-repeat protein locus. Numbers adjacent to nodes indicate bootstrap support greater than 70%.

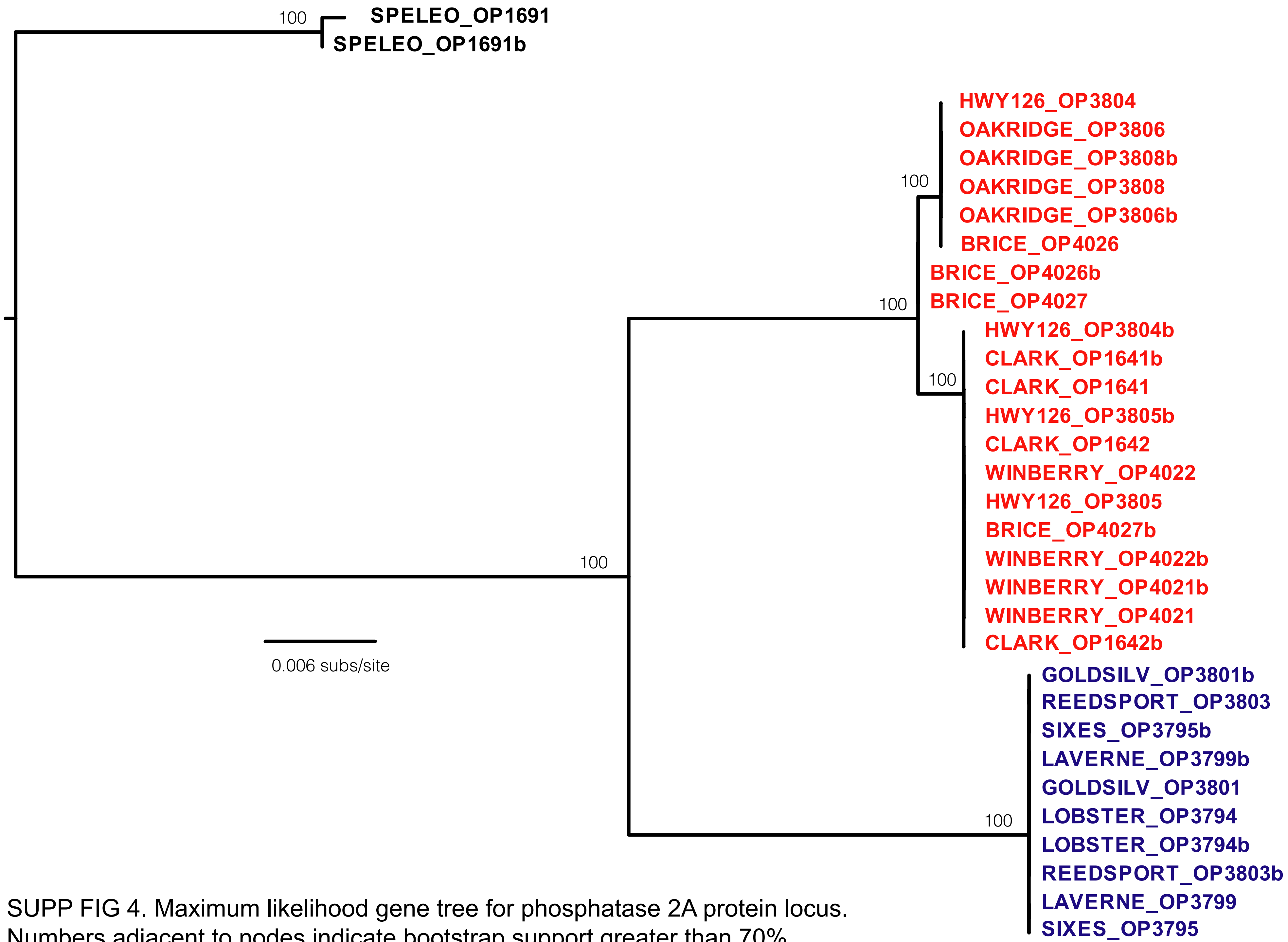

SUPP FIG 4. Maximum likelihood gene tree for phosphatase 2A protein locus. Numbers adjacent to nodes indicate bootstrap support greater than 70%.

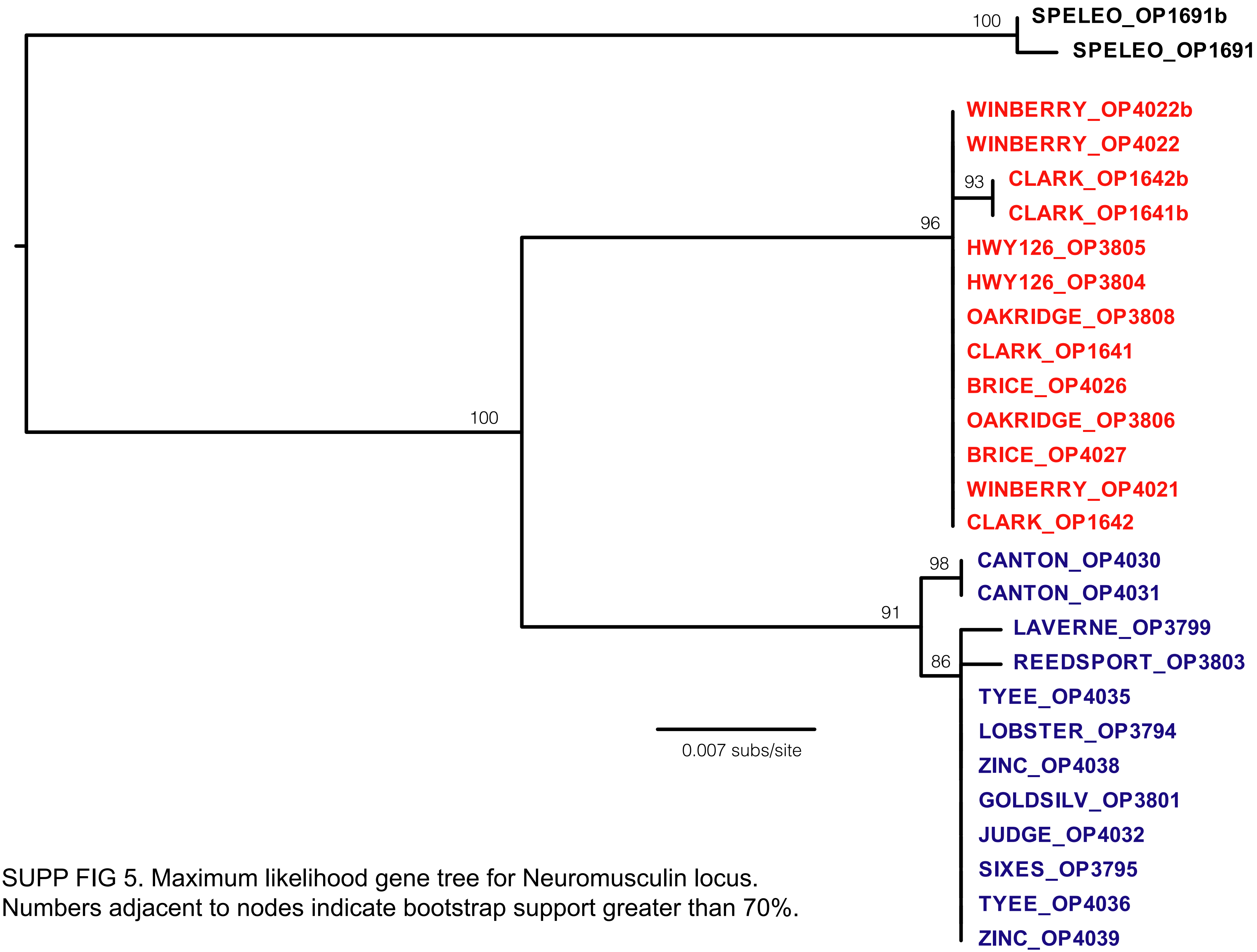

SUPP FIG 5. Maximum likelihood gene tree for Neuromusculin locus.
Numbers adjacent to nodes indicate bootstrap support greater than 70%.
